# Emergence of circulating influenza A H3N2 viruses with genetic drift in the matrix gene: be alert of false‐negative test results

**DOI:** 10.1111/apm.13262

**Published:** 2022-08-10

**Authors:** Rikke Lind Jørgensen, Christian Johann Lerche, Martin Schou Pedersen, Nikolai Soren Kirkby, Amanda Bolt Botnen, Ramona Trebbien, Stephen Nilsson‐Møller, Mette Pinholt, Alex Christian Yde Nielsen, Henrik Westh, Jan Gorm Lisby, Uffe Vest Schneider

**Affiliations:** ^1^ Department of Clinical Microbiology, Copenhagen University Hospital Hvidovre Hospital Hvidovre Denmark; ^2^ Department of Clinical Microbiology, Copenhagen University Hospital Rigshospitalet Copenhagen Denmark; ^3^ National Influenza Center Statens Serum Institut Copenhagen Denmark; ^4^ Institute of Clinical Medicine University of Copenhagen Copenhagen Denmark

**Keywords:** Assay, diagnostic, genetic drift, H3N2, M gene, mutations, RT‐PCR, sequencing, surveillance

## Abstract

In March 2022, we observed samples with a negative fluorescent signal (60.5%, n = 43) for the influenza A matrix gene and a stronger positive signal for subtype A(H3N2). Forty‐three samples were positive in InfA (H3N2) (mean Cq 30.9, range 23.9–35.1), and 26 of the 43 samples were negative in InfA matrix (mean Cq 28.0, range 23.2–30.6). Our multiplex test is a laboratory‐developed four‐target, four‐color influenza A reverse‐transcription PCR assay targeting the matrix gene, subtypes A(H3N2) and A(H1N1)pdm09. Several samples were negative when retested on commercial influenza Point‐of‐Care assays. As the matrix gene is a stand‐alone target in most commercial diagnostic assays, we caution against false‐negative subtype A test results.

## INFLUENZA A

Influenza A virus has a seasonal circulation in humans and a broad host range. Besides causing infections in humans, influenza is also prevalent in other mammals species such as pigs (swine influenza), horses (equine influenza), and avian influenza virus, which can infect poultry and wild migrating birds and aquatic birds, the latter considered to be a natural reservoir for influenza A virus (1). The M gene encodes both matrix and membrane proteins including the matrix protein M1 (nucleotide position 26–784) and membrane protein M2 (nucleotide position 26–51 and 740–1007) (2). The M gene is involved in determining host tropism (3) and is considered to be highly genetically conserved among influenza A viruses (4), making it the chosen target for most clinical real‐time reverse‐transcription polymerase chain reaction assays (RT‐PCR) for detection of influenza A. Currently, circulating subtypes in the human population are A(H3N2) and A(H1N1)pdm09. Influenza A virus causes seasonal epidemics by antigenic drift, and pandemics can be the result of antigenic shift.

## NEGATIVE OR WEAK DETECTION OF THE MATRIX (M1) GENE OF INFLUENZA A

In early March 2022, during an increasing incidence of influenza A in Denmark, clearly positive subtype A(H3N2) clinical patient samples with simultaneous negative or weak detection of the matrix gene (M1 protein) were noticed at the Department of Clinical Microbiology, Copenhagen University Hospital, Hvidovre, Denmark (Table 1). Our laboratory‐developed test (LDT) for influenza A/B diagnostic is designed as a two‐vial assay with four targets and four fluorescent probes for detection of influenza A matrix gene, subtype A(H3N2), A(H1N1)pdm09, and phocine distemper virus (PDV, process control) and three targets and three fluorescent probes for detection of influenza B matrix gene, nucleoprotein (NP), and PDV (Table 2). In 43 InfA H3N2 positive samples (mean Cq 30.9, range 23.9–35.1), we observed missing detection of the matrix gene in 60.5% of samples (n = 26). For positive samples of infA matrix (n = 17), the mean Cq was 28.0 (range 23.2–30.6) and the fluorescence intensity signal of infA matrix was low (n = 7, mean Cq 29.1), medium (n = 6, mean Cq, 28.6), and high (n = 4, mean Cq 25.2), respectively (Table 1).Influenza specimens with RT‐PCR cycle quantification (Cq) of value ≤38 were considered positive for influenza A. Several of the influenza A‐positive samples were retested on various commercially available Point‐of‐Care (PoC) and central laboratory‐based platforms as reported in Table 1. Six of the ten assays reported negative influenza A results in the analyzed patient samples (5–6‐fold dilution).

**Table 1:** Influenza diagnostics on various platforms and assays

Platform/assay	Roche flow LDT multiplex influenza A/B assay (Hvidovre Hospital, Denmark)								
Target	InfA H3N2 (Cq)	InfA H3N2		InfA matrix (Cq)	InfA matrix				InfA H1N1 (Cq)
		Fluorescence			Fluorescence^(ii)^				
		Detected	None‐detected		Low	Medium	High	Neg	
Mean Cq (range)	30.9 (23.9–35.1)			28.0 (23.2–30.6)	29.1 (28.5–30.6)	28.6 (27.2–29.4)	25.2 (23.2–26.8)	–	
Samples (positive/negative)	43/0	43	0	17/26	7	6	4	26	0/43

Platform	Roche Cobas 6800/8800	Roche Cobas Liat	Roche Cobas Liat	Roche Cobas omni Utility	QIAGEN QIAstat‐Dx	Cepheid GeneXpert	Hologic Panther System/Fusion	BioFire Film‐Array	DiaSorin Simplexa
Assay	SARS‐CoV‐2 + Influenza A/B	SARS‐CoV‐2 + Influenza A/B	Influenza A/B + RSV	Influenza A/B + RSV	Respiratory SARS‐CoV‐2 Panel	Influenza A/B + SARS‐CoV‐2 + RSV	SARS‐CoV‐2/Flu or Flu A/B/RSV	RP2.1 plus	Influenza A/B + RSV
Targets InfA	Matrix	Matrix	Matrix	Matrix	Matrix, H1 H3, H1‐2009	Matrix, PB2, PA	Matrix	Matrix, H1, H3, H1‐2009	Matrix
Mean Cq (range)	37.0 (32.8–40.5)	31.9 (26.0–37.0)	32.1 (29.3–36.4)	35.0 (33.6–36.3)	n.a.	n.a.	34.0 (29.2–39.1)	n.a.	32.1 (28.3–35.8)
Samples^(i)^ (Positive/Negative)	3/2	15/0	4/1	2/3	4/4	6/0	14/0^(iii)^	2/3	3/2

N.a., Cq not accessible from manufacturer; Neg, negative; PA, acidic protein; PB2, basic polymerase; RP2.1, Respiratory 2.1 plus Panel.

(i) Positive samples were diluted approximately 5–6‐fold to run multiple platforms. (ii) Inf. A matrix fluorescence in arbitrary units ≤3000 = low, 3001–9999 = medium, ≥10,000 = high. (iii) Fourteen samples were analyzed both by SARS‐CoV‐2/Flu and Flu A/B/RSV assay on Hologic Panther System and Fusion, respectively. Mean Cq and Cq range is shown for the samples analyzed by Flu A/B/RSV on Hologic Panther Fusion system. Positive results for samples analyzed by the SARS‐CoV‐2/Flu assay on Hologic Panther System are accompanied by TTime values (data not shown), positive/negative = 14/0.

**Table 2:** Primers and probes used for detection of influenza A/B in the multiplex LDT assay at Hvidovre Hospital, Denmark

Target	Name	Oligonucleotide sequence. 5′→3′	5′‐end	3′‐end	Final concentration in PCR mix (nM)	Position
Influenza A matrix gene	InfA M Forward primer	CTTCTAACCGAGGTCGAA			500 nM	32–49
(M1 protein)	InfA M Reverse primer 1	TGGTCTTGTCTTTAGCCACTCCAT			250 nM	152–175
RefSeq = NC_007367.1	InfA M Reverse primer 2	TGGTCTTGTCTTTAGCCATTCCAT			250 nM	152–175
	InfA M Probe	TCAGGCCCCCTCAAAGCCGAGAT	LC610	BBQ	250 nM	74–96
Influenza A H3N2 gene	H3N2 Forward primer	GCAACGCTGTGCCTTGG			500 nM	108–124
(Hemagglutinin (H3N2))	H3N2 Reverse primer	AGYTCAGTAGCATTAGTAACTTCAATT	INA		400 nM	176–202
RefSeq = NC_007366.1	H3N2 Probe	CCAAACGGAACGATAGTG	FAM	MGB	150 nM	138–155
Influenza A H1N1 gene	H1N1 Forward primer	TGGACAGGRATGGTAGATGGA			500 nM	1072–1092
(Hemagglutinin (H1N1))	H1N1 Reverse primer	CTTTGTTAGTAATCTYGTCAATGGCAT			700 nM	1160–1186
RefSeq = NC_026433.1	H1N1 Probe	TCAGGRTATGCAGCCGAC	VIC	MGB	200 nM	1126–1143
Influenza B matrix gene	InfB M Forward primer	TCGCTGTTTGGAGACACAATTG			200 nM	4–25
(M1 protein)	InfB M Reverse primer	CAAGGCAGAGTCTAGGTCAAATTCT			700 nM	105–129
RefSeq = OL354958.1	InfB M Probe	CTTTCATTGACAGAAGATGGAGA^(i)^	FAM	MGB	250 nM	34–56
Influenza B NP gene	InfB NP Forward primer	CGCAATTATTCTTCATGTCTTGCT			200 nM	1145–1168
(Nucleoprotein gen)	InfB NP Reverse primer	TGAATTCTGTGCCTGTTAATGCA			500 nM	1203–1225
RefSeq = OL354960.1	InfB NP Probe	CCTATGAAGACCTGAGAGTT	VIC	MGB	250 nM	1178–1197
PDV	PDV Forward primer	CCGTCAAAAGCCGTGATTG			200 nM	3175–3193
	PDV Reverse primer	TGAGTAGGGAGAGCATGTTTTGTT			700 nM	3233–3256
RefSeq = NC_028249.1	PDV Probe	CAGATCAAGCAAGGTTGACCAAAGCCA	Cy5	BBQ	200 nM	3204–3230

BBQ, dark quencher is a non‐fluorescent chromophore; BHQ, Black Hole Quencher; FAM, fluorescein amidites; INA, intercalating nucleic acids; MGB, minor groove binder; Nova, internally placed Nova‐quencher; NP, nucleoprotein; PDV, phocine distemper virus; Y is dC or dT, K is dG or dT, V is dA, dC or dG.

(i) Mismatch of Reference sequence (Ref.Seq). Nucleotide positions are indicated as location in the coding domain sequences for the following GenBank sequences with accession no.: NC_007367.1; Influenza A virus (A/New York/392/2004(H3N2)) segment 7, complete cds: NC_007366.1; Influenza A (A/New York/392/2004(H3N2)) segment 4, complete cds: NC_026433.1; Influenza A virus (A/California/07/2009(H1N1)) segment 4 hemagglutinin (HA) gene, complete cds: OL354958.1; Influenza B virus (B/Egypt/303OP/2018) segment 7 matrix protein 1 (M1) and BM2 protein (BM2) genes, complete cds: OL354960.1; Influenza B virus (B/Egypt/303OP/2018) segment 5 nucleoprotein (NP) gene, complete cds: NC_028249.1; Phocine distemper virus strain PDV/Wadden_Sea.NLD/1988, complete genome.

**Fig. 1:**
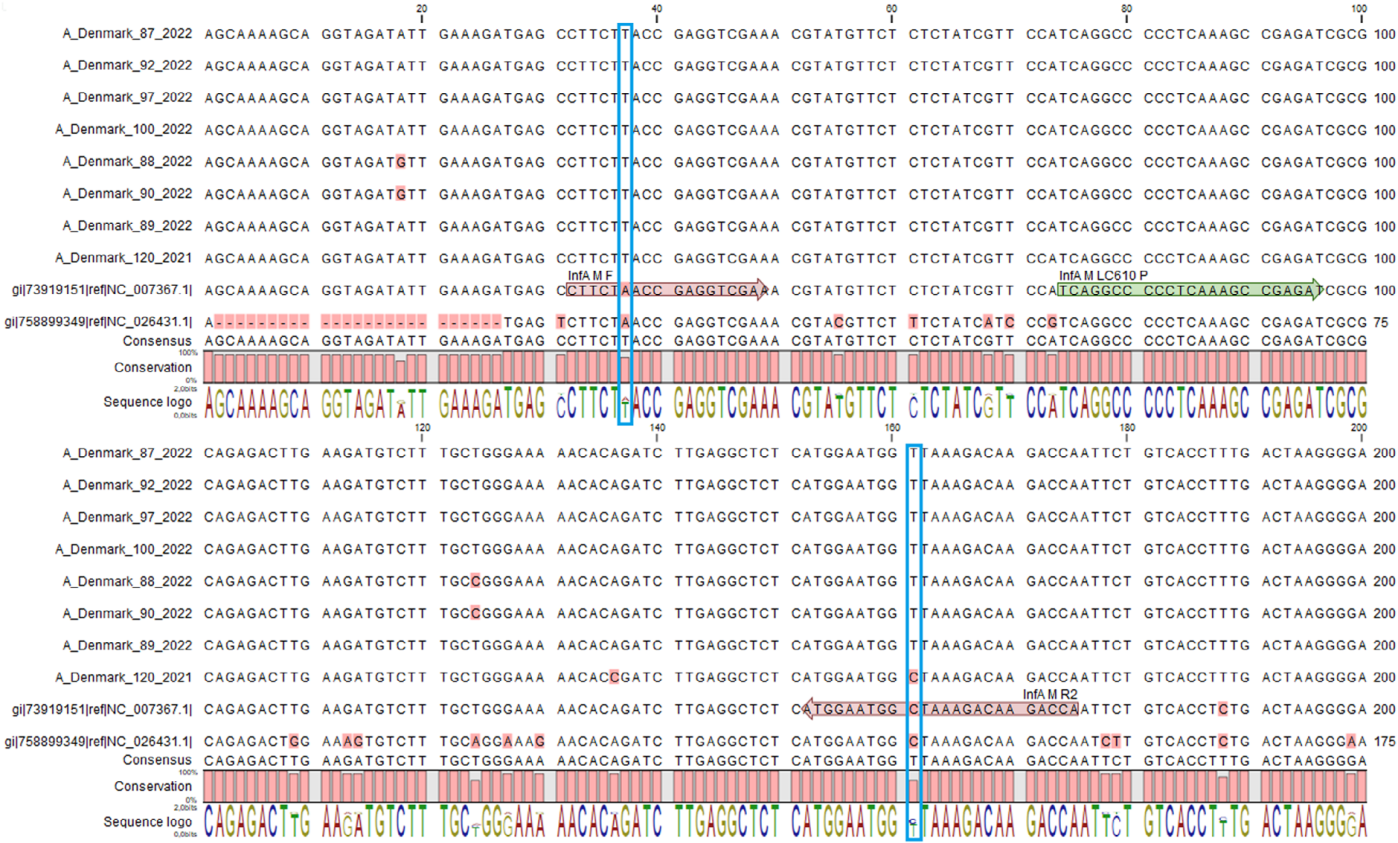
Section (nucleotide position 1–200) of an alignment showing LDT primer and probe binding sites in the influenza A M gene. Alignment of the M gene from seven different circulating influenza A variants belonging to clade 3C.2a1b.2a.2 and one circulating influenza A variant belonging to clade 3C.2a1b.1a (“A_Denmark_120_2021”) was performed by use of the CLC Main WorkBench 21.0.5 software (QIAGEN). NC_007367.1 was used as reference sequence for A(H3N2) and NC_026431.1 as reference sequence for A(H1N1) (https://www.ncbi.nlm.nih.gov/genbank/). Primer binding sites are marked with a red arrow, and the probe binding site is marked with a green arrow. Mutations in the primer binding sites are highlighted with a blue square.

This led to the suspicion of mutational changes in the matrix gene of subtype A (H3N2) within the target regions of our primers and probes. To investigate for possible mutations of the matrix gene, we retrieved eight representatives of circulating subtype A(H3N2) sequences from the Danish National Reference and Surveillance Laboratory for Influenza, Statens Serum Institut (SSI) (GISAID (www.GISAID.org) isolate IDs: A_Denmark_120_2021, A/Denmark/87/2022 EPI_ISL_1080352, A/Denmark/88/2022 EPI_ISL_10803082, A/Denmark/89/2022 EPI_ISL_10802906, A/Denmark/90/2022 EPI_ISL_10803038, A/Denmark/92/2022 EPI_ISL_10803071, A/Denmark/97/2022 EPI_ISL_10803256, A/Denmark/100/2022 EPI_ISL_10803069). Based on these data, we performed alignment of the sequences of our forward and reverse primers and probes for detection of the influenza A matrix gene (Fig. 1).

This alignment revealed a centrally placed single base transversion in the forward primer (A→T), a centrally placed transition in the reverse primer (C→T) for detection of the matrix gene for sequences from 2022 (clade 3C.2a1b.2a.2) and one mutation in the forward primer for sequence 2021 (clade 3C.2a1b.1a) (Fig. 1). No mutations in the probe sequence were found. These findings may explain our observed reduced or missing detection of the influenza A matrix gene due to reduced RT‐PCR efficacy inferred by the two mutations in clade 3C.2a1b.2a.2.

Within the following week, we found 21.3% of all our subtype A(H3N2) positive samples to be negative for the influenza A matrix gene and additionally 30.1% to have reduced detection (low and medium) of the influenza A matrix gene (Table 3).

**Table 3:** Positive influenza samples collected over a seven‐day period

Day	Positive samples^(i)^	Inf. A matrix fluorescens^(ii)^			
		High	Medium	Low	Negative
1	85	40	16	6	23
2	67	30	16	3	18
3	69	30	15	10	14
4	70	35	16	5	14
5	16	9	4	1	2
6	20	14	4	1	1
7	62	31	15	5	11
Mean Cq		25.3	28.1	28.7	–
Total	389	189	86	31	83
% Positive		48.6	22.1	8.0	21.3

(i) All samples shown in the table have positive detection for Inf. A H3N2. (ii) Inf. A matrix fluorescens in arbitrary units ≤3000 = low, 3001–9999 = medium ≥10,000 = high.

We performed additional Sanger sequencing of 10 positive influenza A samples with either a high (n = 5) or negative signal (n = 5) of the matrix gene. The matrix gene was successfully sequenced in eight (high signal, n = 4 and negative signal, n = 4) of the 10 samples (data not shown). The sequencing data of the matrix gene were identical in all eight samples and revealed the same two mutations as shown in Fig. 1.

## LATE INFLUENZA EPIDEMIC IN DENMARK FOR THE 2021–2022 SEASON

Recently, an increase in laboratory‐confirmed cases of influenza has been reported in Denmark (5). In week 12, 12,063 individuals were tested with a positivity rate of 27.3% and an incidence of 56.1 per 100,000 inhabitants (6). The highest positivity rate was seen for week 12 and is now declining (7). The main group affected in this influenza season are young children from 0 to 14 years and elderly above 75 years of age (7). A positivity rate at this level has not been seen since the 2017/18 season, and historically, the influenza peak is most commonly seen around week six. For the season 2021/2022, 1,709,400 individuals (29% of the Danish population) have been influenza vaccinated (7). Noteworthy, the test activity has increased at least twofold during the current influenza season compared with previous years (7).

## DISCUSSION

Among our recent influenza A‐positive clinical patient samples, we have observed a limited detection rate of the matrix gene with our matrix primers, which is an essential target for influenza A diagnostic assays. Due to our dual‐target, dual‐color assay design strategy, we have been able to identify positive subtype A(H3N2) clinical patient samples that would potentially have been diagnosed as false negative for influenza A in assays using, for example, a single target within the influenza A matrix gene as most of the commercial assays (Table 1). It would, therefore, be preferable to use commercial assays at hospitals with more than one target besides the matrix gene for influenza diagnostics.

Our LDT multiplex influenza A/B assay, the SARS‐CoV‐2 + influenza A/B assay (Cobas Liat, n = 15), SARS‐CoV‐2 + Flu (Hologic Panther System, n = 14) and Flu A/B/RSV assay (Hologic Panther Fusion n = 14) and the influenza A/B + SARS‐CoV‐2 + RSV assay (GeneXpert, n = 6) were able to correctly identify all tested positive samples for influenza A. However, we observed “drop outs” for SARS‐CoV‐2 + influenza A/B (Roche Cobas, 3 of 5 positive), influenza A/B + RSV (Roche Liat, 4 of 5 positive), influenza A/B + RSV (Roche Cobas omni Utility, 2 of 5 positive), Respiratory SARS‐CoV‐2 Panel (QIAGEN QIAstat‐Dx, 4 of 8 positive), Respiratory panel 2.1 plus (BioFire Film‐Array, 2 of 5 positive), and influenza A/B + RSV (DiaSorin Simplexa, 3 of 5 positive) indicating potential diagnostic problems. Based on the Cq values of the original patient samples, we expected all diluted samples to be tested positive for influenza A.

Sequencing of eight positive samples with either high (n = 4) or negative (n = 4) detection of the matrix gene revealed no mutational changes in the matrix gene between these two groups as a possible explanation for the reduced detection of the gene. The most likely explanation for the reduced sensitivity of the matrix gene would be due to a lower PCR efficiency caused by the two single base changes located in the center of our matrix primers. Importantly, these changes in the well‐conserved region of the matrix gene will affect several commercial assays using the same target and primers of the matrix gene.

The primers and probes recommended by WHO and CDC for detection of the Influenza A M gene show mutation sites in several primers (see Table S1) after alignment of the eight representatives of circulating subtype A(H3N2) sequences from the Danish National Reference and Surveillance Laboratory for Influenza, Statens Serum Institut (SSI). Therefore, laboratory using these recommendations should be aware of possible pitfalls in diagnostic of influenza A as we observed for a subgroup of circulating influenza A (H3N2).

More clinical samples and a limit of detection study are of course needed to verify this issue and are currently in progress. The reliability of commercially available assays to detect any circulating influenza A or B subtype is of utmost importance for diagnosis and subsequent clinical management of patients suspected for influenza. The increased use of automated molecular systems and PoC “black box systems” challenge the possibility for troubleshooting in general. These PoC systems obviously have their advantages (8, 9) and limitations (10).

For 790 genetically characterized A(H3N2) viruses in the 2021/22 influenza season, the dominating clade has been 3C.2a1b.2a.2 (98% of 790) and the remaining belonged to clade 3C.2a1b.1a in the European Region (11).

In the last two years, the seasonal influenza prevalence has been neglectable, most likely due to the dominance of SARS‐CoV‐2 and the COVID‐19 infection control interventions and restrictions implemented in Denmark. Strategies to detect, prevent, and control influenza are especially important during epidemics and even more in pandemic seasons to reduce overburdening of the healthcare systems.

Early diagnosis and antiviral treatment are crucial, especially for high‐risk patients, to improve outcomes and reduce the risk of complications. The most effective prevention of high burden of the seasonal influenza epidemic is annual vaccination (12). Both national and international surveillance systems are important to alert of any new circulating clades—not only for the purpose of vaccine development, but equally important for clinical diagnostics. Monitoring the genetic drift of circulating viruses (and other microorganisms) and updating primer and/or probes is necessary to ensure continued reliable performance of diagnostic RT‐PCR assays.

## CONCLUSION

In conclusion, our LDT multiplex influenza A/B assay and four commercial assays were able to detect a new circulating strain of A(H3N2) with genetic drift of the matrix gene, while six other commercially available diagnostic assays showed decreased sensitivity for detection of A/H3N2. Hospitalized patients suspected of influenza and testing negative on a matrix gene‐based assay should, therefore, be retested by an alternative influenza assay to ensure correct diagnosis of the current circulating strain of A(H3N2).

## CONFLICT OF INTEREST

None declared.

## Funding information

No specific funding was received. The study was performed as part of routine work at the Departments of Clinical Microbiology.

## Supporting information


**Table S1** Recommended primers and probes used for detection of influenza A/B by WHO/CDC
