# Supplementary material for: Emergence of circulating influenza A H3N2 viruses with genetic drift in the matrix gene: be alert of false‐negative test results
Source: APMIS. 2022 Aug 10;130(10):612–7. doi: 10.1111/apm.13262 (PMC9544743; doi:10.1111/apm.13262)
Supplement: Supplementary file 1 — Table S1 Recommended primers and probes used for detection of influenza A/B by WHO/CDC [file APM-130-612-s001.docx]

**Supplemental Table 1.**

**Recommended primers and probes used for detection of influenza A/B by WHO/CDC**

| **Target** | **Name** | **Oligonucleotide sequence. 5`->3´** |
| --- | --- | --- |
| **WHO/CDC: Protocols_influenza_virus_detection_feb_2021a^(i)^** | | |
| ***Conventional RT-PCR protocols*** | | |
| **InfA Matrix^1^** | M30F2/08  M264R3/08 | ATGAGYCTTYTAACCGAGGTCGAAACG  TGGACAAANCGTCTACGCTGCAG |
| **InfA Full gene^2^** | InfA MF1  MR1027 | AGCAAAAGCAGGTAGATATTGAAAGA  AGTAGAAACAAGGTAGTTTTTTACTC |
| ***Real-time RT-PCR protocols:*** | | |
| **InfA Matrix^3^** | FLUAM-7-F  FLUAM-161-R  FLUAM-49-P6 | CTTCTAACCGAGGTCGAAACGTA  GGTGACAGGATTGGTCTTGTCTTTA  CFO560-TCAGGCCCCCTCAAAGCCGAG-BHQ1 |
| **InfA Matrix^4^** | MP-39-67For  MP-183-153Rev  MP-96-75ProbeAs | CCMAGGTCGAAACGTAYGTTCTCTCTATC  TGACAGRATYGGTCTTGTCTTTAGCCAYTCCA  FAM-ATYTCGGCTTTGAGGGGGCCTG-MGB |
| **InfA Matrix**  **(Flu A)^5^** | InfA Forward  InfA Reverse  InfA Probe | GACCRATCCTGTCACCTCTGAC  AGGGCATTYTGGACAAAKCGTCTA  FAM-TGCAGTCCTCGCTCACTGGGCACG_BHQ1 |
| **InfA Matrix^6^** | AM Forward  AM Reverse  AM Probe | GAGTCTTCTAACMGAGGTCGAAACGTA  GGGCACGGTGAGCGTRAA  JUN - TCAGGCCCCCTCAAAGCCGAG - QSY |
|  | | |
| **CDC Flu SC2 Multiplex Assay^(ii)^** | | |
| **InfA Matrix** | InfA Forward 1  InfA Forward 2  InfA Reverse 1  InfA Reverse 2  InfA Probe | CAAGACCAATCYTGTCACCTCTGAC  CAAGACCAATYCTGTCACCTYTGAC  GCATTYTGGACAAAVCGTCTACG  GCATTTTGGATAAAGCGTCTACG  FAM/TGCAGTCCT/Nova/CGCTCACTGGGCACG/BHQ-1 |

Mismatch to the eight representatives of circulating subtype A(H3N2) sequences from the Danish National Reference and Surveillance Laboratory for Influenza, Statens Serum Institut (SSI) are marked with red color.

^(i)^http://www.who.int/csr/resources/publications/swineflu/CDCrealtimeRTPCRprotocol_20090428.pdf

^(ii)^CDC Flu SC2 Multiplex Assay: <https://www.cdc.gov/coronavirus/2019-ncov/lab/multiplex-primer-probes.html>

^1^ Primers and probes used for detecting influenza A in Annex 1, A.: Protocol 1

^2^ Primers and probes used for detecting influenza A in Annex 1, D.: Protocol 1.

^3^ Primers and probes used for detecting influenza A in Annex 2, A.: Protocol 1 and Protocol 2, and Annex 4: Protocol 1

^4^ Primers and probes used for detecting influenza type A viruses (M gene) in Annex 2, D.: Protocol 3.

^5^ Primers and probes used for detecting influenza A (Gene Matrix) CDC protocol 2009, Annex 4: Protocol 1.

^6^ Primers and probes used for detecting influenza A (M gene) in Annex 4: Protocol 3.
